# Prevalence of Anemia, Overweight/Obesity, and Undiagnosed Hypertension and Diabetes among Residents of Selected Communities in Ghana

**DOI:** 10.1155/2017/7836019

**Published:** 2017-08-15

**Authors:** Alex Kojo Anderson

**Affiliations:** Department of Foods and Nutrition, University of Georgia, Athens, GA 30602, USA

## Abstract

The increasing numbers of lifestyle related chronic diseases in developing countries call for awareness, early detection, and effective management. The objective of this paper is to report the prevalence of undiagnosed hypertension, diabetes, overweight/obesity, and anemia among residents of selected communities in Ghana. The data comes from a community screening conducted in Ghana as part of the University of Georgia Summer Service Learning Program. Descriptive statistics were used to summarize the data while chi-square and independent *t*-test compared groups. A total of 976 participants (37.9% males and 62.1% females), 18 years and older, were screened. Mean age was 46.25 ± 17.14 years, BMI was 25.44 ± 5.26 kgm^−2^, and hemoglobin was 12.04 ± 2.22 g/dL. 3.1% and 12.6% reported existing diagnosis for diabetes and hypertension, respectively. Almost half (47.8%) were overweight/obese; 27.0% were hypertensive while 34.0% had diabetes. Also, 28.8% males compared to 37.8% females had diabetes (*P* = 0.015), while 28.2% males compared to 26.2% females were hypertensive (*P* = 0.635). There were differences in BMI (*P* < 0.0001), anemia (*P* = 0.007), and undiagnosed diabetes (*P* < 0.0001) and hypertension (*P* < 0.0001) by community (Takoradi versus Cape Coast) where the screening took place. Findings from the screening exercise call for improved public health education with a focus on lifestyle habits and health seeking behaviors among Ghanaians.

## 1. Introduction

Hypertension (raised blood pressure), one of the major diseases of the 21st century, is reported to affect over 1 billion of the world's population with about three-fourths living in developing countries and almost half (46%) in Africa [1–3]. Hypertension is also a major risk factor for a number of chronic diseases including heart disease, heart attack/failure, stroke, cerebrovascular disease, diabetes mellitus, and renal failure. Unfortunately, because of the asymptomatic nature of hypertension during the early stages of the disease, it has not been a priority for primary healthcare workers, particularly in developing countries with limited resources and poor healthcare infrastructure.

Type 2 diabetes (due to insulin insensitivity and/or poor blood glucose management) currently affects 1 in 11 adults, and a predicted 642 million people are expected to be living with diabetes in the world by the year 2040 [4, 5]. Recent reports suggest that almost 3 of every 4 diabetics reside in low- and middle-income countries, expected to further increase by the year 2030 [6, 7]. Poorly managed diabetes increases the risk of cardiovascular disease, blindness, kidney failure, amputations, and early death. It is projected that the prevalence of diabetes in Africa will increase and surpass those of the other regions of the world by the year 2035 [8–10].

Anemia (low blood hemoglobin concentration) is one of the significant public health problems (hidden hunger) plaguing the world today [11]. Globally, over 1.6 billion people are reported to be anemic [12]. Anemia occurs through the lifecycle but disproportionately affects children and women and individuals from low-income, resource limited areas of the world. Although iron deficiency is the significant cause of anemia, there are other contributing causes of anemia which include parasitic infections, malaria, heavy blood loss as a result of menstruation among females, and other nutrient deficiencies including folate, vitamin B_12_, riboflavin, and copper. Because of the silent nature of anemia, it often goes undiagnosed and poorly managed, leading to poor cognitive and physical development of children and negative physical performance and work productivity among adults [13].

The prevalence and risk of death from hypertension and diabetes are on the increase in most developing countries as a result of the ongoing nutrition transition and poor health infrastructure in these countries [8, 14–23]. The impact of the nutrition transition on the dietary habit is the resulting change from traditionally less processed foods to Western food habits and lifestyles which include the consumption of foods high in calories, sugar, salt, and total fat [24, 25]. These dietary changes have led to increased prevalence of obesity, metabolic syndrome, diabetes, and hypertension in most low- to middle-income countries [26, 27]. This is compounded by poor health seeking behaviors, poor healthcare infrastructure and resource allocation, and inadequate access to preventative health services. Because of the limited healthcare resources, governments of developing countries, including Ghana, tend to focus most of their effort and resources on infectious diseases and not chronic diseases [19, 28]. Another major problem is the lack of awareness and magnitude of these chronic diseases and their early detection and intervention among populations of developing countries, including Ghana [26]. Thus, in this paper we report the prevalence of undiagnosed hypertension and diabetes, overweight/obesity, and anemia among residents of selected communities in Ghana and their public health implications.

## 2. Methods

### 2.1. Study Design and Setting

This was a secondary data analysis of data generated from a community nutrition and health screening/education which is part of service learning activities conducted by students from the University of Georgia who participated in a Service Learning Program in Ghana directed by the author. The community nutrition and health screening/education was conducted between June and July 2016. Participants were free living adult community residents who came forward to participate in the screening exercise. The use of the data in this study was approved by the Human Subject Institutional Review Board of the University of Georgia.

The community nutrition and health screening/education exercises occurred in Sekondi-Takoradi, capital city of the Western Region and Cape Coast, capital city of the Central Region of Ghana. The mobile clinics for the nutrition and health screening/education were in two (2) communities (Collins Avenue and Star of the Sea Cathedral) in Takoradi and Apewosika community in Cape Coast.

Sekondi-Takoradi is located at the southeastern part of the Western Region. According to the 2010 Population and Housing Census, the total population of the Sekondi-Takoradi is 559,548 (23.5% of the region's total population). Over 67% of the population are 15 years and older. Over 96% of the population in Sekondi-Takoradi resides in urban areas. Almost 91% of the population in the Metropolis are literate (having some form of formal education) while 90% of the adult population are employed. Health facilities include the regional hospital, 2 district hospitals, and a number of clinics that provide healthcare services to residents of the area and other neighboring communities.

The total population of Cape Coast is 169,894 (constituting about 7.7% of the total population of the Central Region) of which over 120,000 are 15 years and older. Cape Coast is predominantly urban with three-quarters of the population residing in urban areas. Over 90% of the population of Cape Coast is literate compared to 78.2% and 74.1% for the Central Region and Ghana, respectively. Almost 91% of adults are employed. Cape Coast is the home of the regional hospital which also serves as a referral center. It also has a district hospital and a number of clinics that provide healthcare to the population of the Metropolis.

### 2.2. Data Collection Procedures

Data for this study was generated from adults 18 years and older who voluntarily participated in the University of Georgia Ghana Summer Service Learning from June to July 2016. The data available included community where the screening occurred, age, gender, weight, height, blood hemoglobin, fasting blood glucose, and blood pressure.

### 2.3. Anthropometric Measurement

Weight and height were measured using calibrated weighing scale and stadiometer following standard protocol [29]. Because these anthropometric measurements were conducted in the open, the requirement for minimum clothing during weight measurement was not possible. Weight was measured with participants wearing their regular clothing devoid of other clothing accessories in the case of women and wallets and so forth in the pocket of men. Weight was measured to the nearest 0.1 kg. Height was measured to the nearest 0.1 cm without shoes, cap, or headgear. Weight and height measurements were used to estimate body mass index (BMI = weight in kg ÷ [height in meters]^2^). BMI was further classified as underweight (BMI < 18.5 kgm^−2^), normal weight (BMI = 18.5–24.9 kgm^−2^), overweight (BMI = 25.0–29.9 kgm^−2^), and obese (BMI ≥ 30.0 kgm^−2^).

### 2.4. Hemoglobin

Blood hemoglobin level was measured using HemoCue Hb201^+^. To measure blood hemoglobin level, the patient's forefinger was prepared by wiping the tip with alcohol to sterilize the area and then allowed to dry. The sterilized area was then pricked with a lancet and a drop of blood collected into a microcuvette. The microcuvette filled with the blood was then inserted into the HemoCue Hb201^+^ for the hemoglobin measurement. The measured hemoglobin was classified as normal (Hb > 13 g/dL for men and Hb > 12 g/dL for women), mild anemia (Hb: 11.0–12.9 g/dL for men versus Hb: 11.0–11.9 g/dL for women), moderate anemia (Hb: 8.0–10.9 g/dL), and severe anemia (Hb < 8 g/dL).

### 2.5. Fasting Blood Glucose

Blood glucose concentration was measured using HemoCue Glucose 201^+^. For blood glucose measurement, the patient forefinger was prepared as described for hemoglobin measurement above. A drop of blood was collected from a finger prick into a microcuvette and inserted into the HemoCue Glucose 201^+^ for blood glucose measurement. The measured blood glucose was later classified as normal (FBG < 100 mg/dL), prediabetes (FBG: 100–125 mg/dL), and diabetes (FBG > 126 mg/dL) [30].

### 2.6. Blood Pressure

Blood pressure was measured at least 10 minutes from the patient's arrival at the mobile screening clinic. Participants were seated and made comfortable with one arm resting on a table. The BP was measured using an automated (digital) sphygmomanometer. The cuff was inflated by turning on the sphygmomanometer for the blood pressure measurement. The measured blood pressure was classified as normal (systolic: less than 120 mmHg; diastolic: less than 80 mmHg), prehypertension (systolic: 120–139 mmHg; diastolic: 80–90 mmHg), and hypertension (systolic: ≥140 mmHg; diastolic ≥ 90 mmHg).

### 2.7. Statistical Analysis

The statistical package IBM SPSS Statistics 23 for Windows (IBM SPSS Inc., Armonk, NY, USA) was used for data entry and analysis. Descriptive statistics were used to summarize the data and results reported as percentages (frequencies) and means (± standard deviation). Student's unpaired, two-sided *t*-test was used to compare means between Takoradi and Cape Coast. Pearson Chi-squared test (Fisher's exact test) was used to compare the independent variables (place of resident, age, and gender) and outcome variables (indicators of anemia, obesity, diabetes, and hypertension). All *P* values were two-sided, and criterion for statistical significance was set at *P* < 0.05.

## 3. Results

A total of 976 adults (597 from Takoradi and 379 from Cape Coast) participated in the screening with data for the study. The average age was 46.25 ± 17.14 (range: 18–100) years and was similar in the two Metropolises. Majority of the participants were over 40 years old. Participants were fairly distributed by gender (*P* = 0.437) in the two cities with over 60% being females (Table 1). For individuals who participated in the screening exercise, 12.6% (13.7% in Takoradi versus 10.8% in Cape Coast) and 3.1% (3.7% in Takoradi versus 2.1% in Cape Coast) reported known (existing) diagnosis for hypertension and diabetes, respectively.

### 3.1. Participant Hemoglobin

**Figure 1:**
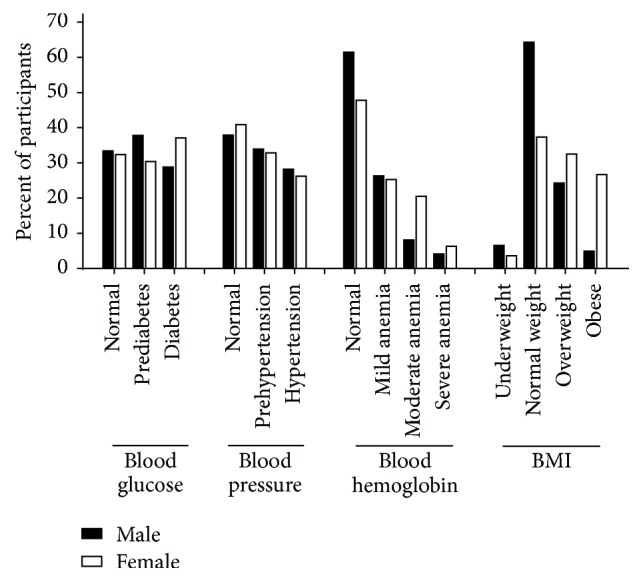
Prevalence of undiagnosed diabetes and hypertension, anemia, and obesity.

The mean hemoglobin level was 12.04 ± 2.22 g/dL (range: 2.00–17.80). There were differences in hemoglobin levels by gender and screening community. The mean blood hemoglobin concentration was significantly lower among females compared to males (11.43 ± 2.17 g/dL versus 13.03 ± 2.17 g/dL, *P* < 0.0001) and Takoradi compared to Cape Coast (11.85 ± 2.38 g/dL versus 12.34 ± 1.92 g/dL, *P* = 0.001), although these were within the normal range. Overall, 47.0% of the participant had mild to severe anemia. About a third of males (30.5%) and a little over half (52.2%) of females had mild to severe anemia according to their blood hemoglobin concentration (Figure 1). Prevalence of anemia was significantly (*P* = 0.007) higher among participants from Takoradi compared to their counterparts from Cape Coast (Table 1).

### 3.2. Participant Weight

Overall, almost half (47.8%) of the participants were either overweight or obese (BMI > 25 kgm^−2^). The average BMI was 26.06 ± 5.32 kgm^−2^ versus 24.45 ± 5.01 kgm^−2^ for participants in Takoradi and Cape Coast, respectively (*P* < 0.0001). 18.4% of the participants were obese. Whereas 53.7% of participants from Takoradi were either overweight or obese, only 38.4% of those from Cape Coast were overweight or obese (*P* < 0.0001). The prevalence of overweight/obesity was higher among female participants compared to male participants (*P* < 0.0001) (Figure 1).

### 3.3. Blood Pressure

Generally, measured systolic and diastolic blood pressures were higher for participants from Takoradi compared to those from Cape Coast. The measured blood pressure shows that majority of the participants were either prehypertensive or hypertensive. The overall prevalence of hypertension was 27.0%. Of those with hypertensive blood pressure, only 12.6% (123/976) had prior diagnosis for hypertension. The prevalence of hypertension detected from the screening exercise was significantly (*P* < 0.0001) higher among participants from Takoradi (30.4%) compared to their counterparts from Cape Coast (21.8%). There was overall significant (*P* = 0.002) difference in hypertension by participant gender, with males having slightly higher prevalence compared to females (Figure 1). Similar trends were observed in Takoradi and Cape Coast (data not shown).

### 3.4. Fasting Blood Glucose

Majority of the participants had fasting blood sugar levels classified as either prediabetes or diabetes. The measured fasting blood glucose showed 39.2% prevalence for diabetes of which 3.1% (30/976) reported known diabetes diagnosis. Almost 40% of participants from Takoradi were diabetics compared to 24.6% from Cape Coast based on their measured fasting blood sugar (*P* < 0.0001) (Table 1). Most (39.8%) of the participants from Cape Coast compared to 28.4% from Takoradi had measured fasting blood sugar level in the normal range. There was statistical difference in fasting blood sugar level by participant gender with 37.1% of females compared to 28.8% of males who were diabetics (*P* = 0.015) (Figure 1).

## 4. Discussion

Ghana, as is the case of many low- and middle-income countries, is dealing with the triple burden of malnutrition (undernutrition, micronutrient deficiencies, and increasing prevalence of overweight/obesity and related chronic diseases) because of changes in their food systems due to their degree of economic development and adopting Western food culture [31]. The situation poses a threat to the healthcare infrastructure and productivity and creates a financial burden for these countries. There is therefore a need to bring attention to awareness and early intervention to mitigate the human resource and economic burden of these countries. In this study, we examined the problem of undiagnosed hypertension and diabetes, overweight/obesity, and anemia among residents of Takoradi and Cape Coast, Ghana. We observed high prevalence of undiagnosed hypertension and diabetes, overweight/obesity, and anemia among residents who participated in the screening exercise.

The findings of the present study show overall prevalence of undiagnosed hypertension and diabetes to be 53.2% (77.3% in Takoradi and 0.0% in Cape Coast) and 90.9% (90.5% in Takoradi and 91.3% in Cape Coast), respectively. Our finding is consistent with findings from a study by Amoah and colleagues conducted in Accra, Ghana, where they reported prevalence of undiagnosed diabetes [32]. However, the prevalence of undiagnosed diabetes among participants in the current study from Takoradi and Cape Coast is higher than reported by Amoah and colleagues [32]. This suggests a lack of awareness or knowledge of symptoms and general education of these diseases among residents of Takoradi and Cape Coast. This is a major public health problem as previous studies have shown that awareness and early detection leads to effective management of hypertension and diabetes [33–36]. This observation paints a bleak health picture for both Takoradi and Cape Coast as this stands to affect the work productivity and quality of life of the adult population of these areas, which will eventually have a negative impact on their economic growth as these adults constitute the workforce. This situation also has the potential of posing a great challenge to the healthcare infrastructure and increasing healthcare costs for the government of Ghana and the individuals involved as well as their respective families.

The prevalence of overweight and obesity was disproportionately higher among participants from Takoradi compared to those from Cape Coast (*P* < 0.0001). Over half of participants from Takoradi compared to about a third from Cape Coast were either overweight or obese. This is higher than previously reported among the adult population in Ghana and other developing countries [37]. For example, a recent systematic review and analysis conducted by Ofori-Asenso and colleagues of 43 studies conducted across the 10 regions of Ghana reported that about 43% of the adult population are either overweight or obese. Women generally had a higher prevalence of overweight/obesity compared to men [37]. This differential and high prevalence rate of overweight/obesity by gender as was observed in the current study has been attributed to cultural perception of body size. In most African countries including Ghana, being overweight or obese is associated with good health, wellbeing, and high social class [20, 23, 38–43]. Prevailing knowledge of the strong association between obesity and chronic diseases including hypertension and diabetes paints a gloomy picture of the health of Ghanaian adults should this continue [44–46]. There is therefore a need for culturally appropriate interventions that target healthy weight management among Ghanaian adults, especially women, so they understand the health risks associated with overweight and obesity and that being overweight or obese is not necessarily an indication of good health status and wellbeing.

The prevalence of anemia observed among participants in this study was similar to that reported in a number of systematic reviews of global level data [11, 12, 47]. This observation is a significant public health problem as anemia has direct effect on one's performance and productivity. In the current study we could not ascertain the cause of the high anemia prevalence due to our inability to obtain information on potential causes and general lifestyle of the participants. The high anemia prevalence could be due to a number of factors including genetic make-up (such as sickle cell trait and thalassemia), dietary habits (micronutrient deficiencies), infections such as hookworm and schistosomiasis, and disease states such as malaria or inflammation [48]. Other evidences suggest increased risk for anemia among patients with diabetes [49]. This is evidential in the current study with high prevalence of undiagnosed diabetes and anemia.

This study comes with a number of limitations which should inform the interpretation of the findings. First, this is a secondary data analysis, and the data used came from a community screening exercise, thus the likelihood of selection bias. Second, we did not have data to examine the predictors of anemia, overweight/obesity, hypertension, and diabetes. Third, the screening exercise took place within communities in Takoradi and Cape Coast; therefore, our findings may not be representative of respective regions limiting the generalizability of the findings; thus results should be interpreted with caution. However, the study had a large sample size, which is a strength, and also highlights the lack of awareness of hypertension, diabetes, overweight/obesity, and anemia in communities in Ghana to inform public health interventions.

## 5. Conclusions

The situation of high prevalence of obesity, anemia, and undiagnosed hypertension and diabetes has significant public health implications for health planners of a country such as Ghana which has limited health infrastructure with a heavy burden of undernutrition and infectious diseases among its population. There is therefore an urgent need for health policies focused on primary prevention and early detection of these chronic disease conditions within the Ghanaian population. The ongoing nutrition and epidemiologic transition and urbanization in Ghana have affected the traditional food culture and general lifestyle. The situation poses a serious threat to the wellbeing, quality of life, and productivity of Ghana's workforce if immediate steps are not taken to reverse these rising trends.

## Figures and Tables

**Table 1:** Characteristics of participants.

	Takoradi	Cape Coast	*P* value
	Mean ± SD	Mean ± SD	
Age (years)	46.45 ± 16.69	45.92 ± 17.86	0.637
BMI (kgm^−2^)	26.06 ± 5.32	24.45 ± 5.01	<0.0001
Systolic blood pressure (mmHg)	136.89 ± 24.80	134.27 ± 25.53	0.114
Diastolic blood pressure (mmHg)	86.98 ± 14.62	81.81 ± 14.04	<0.0001
Fasting blood glucose (mg/dL)	123.46 ± 43.41	112.86 ± 38.37	<0.0001
Hemoglobin (g/dL)	11.85 ± 2.38	12.34 ± 1.92	0.001

	*n* (%)	*n* (%)	

Age (years)			
<25 25–40 41–60 >60	61 (10.2) 177 (29.6) 222 (37.2) 137 (22.9)	45 (11.9) 120 (31.7) 136 (35.9) 78 (20.6)	0.661
Sex			
Male Female	228 (38.2) 369 (61.8)	142 (37.5) 237 (62.5)	0.437
Weight status			
Underweight Normal weight Overweight Obese	21 (3.5) 255 (42.8) 196 (32.9) 124 (20.8)	25 (6.6) 208 (55.0) 90 (23.8) 55 (14.6)	<0.0001
Measured blood pressure			
Normal Prehypertensive Hypertensive	204 (34.2) 211 (35.4) 181 (30.4)	184 (48.5) 113 (29.8) 82 (21.6)	<0.0001
Fasting blood glucose			
Normal Prediabetes Diabetes	169 (28.4) 189 (31.8) 237 (39.8)	149 (39.8) 133 (35.6) 92 (24.6)	<0.0001
Hemoglobin			
Normal Mild anemia Moderate anemia Severe anemia	305 (51.1) 150 (25.1) 98 (16.4) 44 (7.4)	211 (56.1) 100 (26.6) 56 (14.9) 9 (2.4)	0.007
